# Device-measured physical activity and sedentary behavior during office versus remote workdays among hybrid workers

**DOI:** 10.5271/sjweh.4335

**Published:** 2026-11-01

**Authors:** Tuija Leskinen, Miika Tuominen, Kristin Suorsa, Jesse Pasanen, Sanna Pasanen, Vilma Iisalo, Virpi Ruohomäki, Katja Pahkala, Olli J Heinonen, Sari Stenholm

**Affiliations:** 1 Department of Public Health, University of Turku and Turku University Hospital, Finland.; 2 Centre for Population Health Research, University of Turku and Turku University Hospital, Finland.; 3 Finnish Institute of Occupational Health, Helsinki, Finland.; 4 Research Centre of Applied and Preventive Cardiovascular Medicine, University of Turku, Finland.; 5 Paavo Nurmi Centre and Unit for Health and Physical Activity, University of Turku, Finland.; 6 Research Services, Turku University Hospital and University of Turku, Finland.

**Keywords:** accelerometry, office work, sitting, work from home, workplace, remote work

## Abstract

**Objectives:**

Daily sedentary time (SED) has been found to be higher during remote compared to office workdays, but current evidence mostly relies on studies from COVID-19 pandemic times. This study aimed to compare device-measured physical activity (PA) and SED between office and remote workdays, worktimes, and non-worktimes among Finnish hybrid workers after the pandemic.

**Methods:**

Overall, 97 university employees [84% women, mean age 41.6 (standard deviation 10.5) years] wore Fibion SENS sensors on their thigh for seven consecutive days and provided at least one remote and one office workday. SED, light PA (LPA), and moderate-to-vigorous PA (MVPA) were measured for remote and office workdays, worktimes, and non-worktimes. A linear mixed model was used to study the within-individual differences.

**Results:**

Remote workdays had 44 minutes [95% confidence interval (CI) 25–64] higher SED, 29 minutes (95% CI 11-48) lower LPA, and 15 minutes (95% CI 10–20) lower MVPA compared to office workdays. Also, remote worktime included 32 minutes (95% CI 16–48) more SED, 25 minutes (95% CI 9–41) less LPA, and 7 minutes (95% CI 5–9) less MVPA compared to office worktime. Non-working hours had higher SED and lower MVPA during remote versus office workday.

**Conclusions:**

Remote workdays included less PA and more SED compared to office workdays, especially during working hours. Because of the popularity of remote work, strategies to promote PA during remote workdays, in addition to commonly recommended strategies to reduce sitting, are warranted.

Daily physical activity (PA) is accumulated during worktime, leisure time, and household chores. It is well-known that occupation contributes to daily total PA (1). Unlike manual workers, who accumulate most of their daily PA during working hours, office employees spend the majority of their worktime sedentary (2). Sedentary behavior, ie, any waking behavior with an energy expenditure ≤1.5 metabolic equivalents in sitting, reclining, or lying posture (3), is a well-known risk factor for metabolic disturbances (4, 5). Therefore, evidence-based interventions and actions, for example the use of sit-stand desks, digital prompts and reminders, activity breaks, counseling, or a combination of these methods (6–8), have been applied to reduce sitting time in office settings.

After the COVID-19 pandemic, remote work has remained a common way of working (9). According to recent statistics, over one fifth of US citizens (10) and Europeans (11) work at least partly remotely, with Finland having similar percentages for remote work (12). Previous survey-based studies have shown that working from home during the pandemic was associated with increased daily sedentary time (SED) and reduced PA compared with working at the office (13), raising concerns of potential adverse health effects (14). More modern, device-based studies have similarly suggested that working from home is associated with >30-minute higher SED and approximately 2500 fewer steps per day compared to on-site work (15). However, a majority of the evidence still relies on studies conducted during periods of pandemic restrictions. Consequently, post-pandemic evidence is still limited to only few studies (16–18). The higher SED while working from home has been explained by the lack of active commuting and social interaction and limited space in home offices (13). Because of these differences in work environments and daily behaviors between office and home-based work, the existing office-based actions and tools to increase PA or decrease SED may not be applicable for remote work settings (19). Therefore, more studies on and understanding of PA and SED during remote workdays are warranted to inform the development of future interventions and strategies (14).

To date, accelerometer-based studies comparing PA and SED between office and remote workdays among office workers have showed that remote workdays include more in-bed time (20, 21), higher daily SED (16–18, 22), and eg, less daily moderate-to-vigorous PA (MVPA) (18, 23) when compared to office workdays. However, some of these abovementioned studies have been conducted with relatively small groups of office workers (N=17–55) (20, 22, 23) whereas others included larger samples (N=94–177). In addition, the existing findings among office workers are mostly reliant on daily level assessments, which does not allow for distinguishing between PA and SED accumulated during working versus non-working hours. To fill this gap, our objective was to examine whether there are within-individual differences in daily total, worktime, and non-worktime PA and SED between office and remote workdays among Finnish university employees during post-pandemic period (24). Participants had a high degree of flexibility to decide whether to work remotely or at their employer’s premises. Therefore, we refer to them as hybrid workers.

## Methods

The Daily Physical Activity among Hybrid Workers (WORKDAY) study aimed to compare PA and SED between office and remote workdays. Study participants were recruited from the campus area of the University of Turku, university hospital, and University of Applied Sciences, Finland, by advertising the study every other week in the internal staff bulletins and email lists, and with continuous advertisements on campus buildings’ noticeboards. The recruitment began in May 2023 and lasted for one year, excluding the summer holiday and Christmas periods. Eligible participants were those office employees who were able to work both at the office and remotely, ie, engage in hybrid work. A link/QR code for the registration questionnaire was offered in the advertisement. The registration questionnaire included a description of the study with a choice of participation (yes/no). Participating respondents were asked to complete an information sheet and select a preferred measurement week during semesters. The measurements were conducted between May 2023 and June 2024. Of all measurements, 11% were conducted during winter, 43% in spring, 13% in summer, and 32% in autumn.

Overall, 108 persons completed the registration questionnaire, of which 4 declined to participate. Of the 104 participants, 5 dropped out due to scheduling issues, leaving 99 (84% women) available for the measurements. The analytical sample of 97 hybrid workers consisted of participants who provided at least one valid remote workday and one valid office workday: 2 participants had insufficient data.

The Ethics Committee for Human Sciences at the University of Turku approved the WORKDAY study (13/2023). All participants gave their written informed consent before the measurements were taken. A power analysis to determine the minimum sample size of N=88 was calculated for a 30-minute [standard deviation (SD) 99] difference in daily SED according to previous finding (22), with a significance level of 0.05 and desired power of 0.80. The recruitment was extended to up to 100 participants to account for potential 10% drop-out and possible technical malfunctions affecting data collection.

### Measurement of physical activity and sedentary behavior

PA and SED were measured with the Fibion SENS Motion System, which includes a triaxial SENS motion® sensor, a smartphone application and a web-based application that has shown moderate-to-excellent agreement with video observation for the assessment of PA and SED (25). The Fibion SENS sensor records acceleration with a 12.5 Hz sampling frequency, which was set by the manufacturer (25). Research personnel met each participant in person and provided both verbal and written instructions on how to wear the sensor. The participants were instructed to fasten the sensor with an adhesive waterproof film dressing directly to the skin midway on the thigh, approximately 5−10 cm above the knee joint. Thigh-sensor placement was chosen because it allows the separation of sitting/lying from standing and enables the detection of specific PA behaviors with high accuracy (25–27). The participants were asked to wear the sensor continuously for 24 hours per day for ≥7 consecutive days, including during water-based activities but to remove it when in the sauna or in case of skin irritation. The measurement period was requested, but not restricted, to include two office workdays and two remote workdays. The participants were also instructed to complete a daily log during their measurement period to record daily work modes (office, remote, combined, day off), daily working hours, and in- and out-of-bed times. After the measurement period, the participants returned the sensors and daily logs to pointed return boxes located within the University buildings or via mail. The sensor did not provide any feedback to the participants while measuring; however, after data processing, each participant received detailed feedback on their daily SED and a suggestion for actions.

The raw data from the sensors were downloaded via Bluetooth using a smartphone application and transmitted to a secure web server for storage and later analysis. Data were sampled in 5-second epochs and automatically categorized into time spent in the predefined activity categories using a validated built-in algorithm (25). The activity categories included sitting or lying awake as SED (3); standing, sporadic walking, and continuous light walking as light PA (LPA); biking, moderate intensity activity (eg, brisk walking), and high intensity activity (eg, running) as MVPA. Total steps taken were calculated for each epoch.

A valid measurement day was defined a priori as ≥10 hours of sensor wear time during waking hours, which has been a common criterion for a valid measurement day in previous literature (28). Sleep time was estimated using in- and out-of-bed times from daily logs. The non-wear time of the sensor was defined when activity categorization was not possible and excluded using the predefined category called “no data” (25). The mean time spent in different activity categories and the number of steps were calculated for valid office and remote workdays, and separately for working hours (ie, reported worktime when the sensor was worn) and non-working hours (ie, sensor wear time outside the reported working hours and reported in-bed period) within each workday type. Valid workdays including both office and remote worktimes (N=19 days, 4% of all valid workdays) and days off from work, ie, days without reported worktime according to the daily logs, were excluded. The hourly total PA minutes (LPA+MVPA) was used to illustrate the pattern of PA during typical waking hours (6–23 hours) for both office and remote workdays.

### Participant characteristics

Age, sex, occupational title, mean weekly working hours, self-rated health, self-reported height and weight, living alone (yes/no), and the number of children <18 years old were acquired with a web-based study questionnaire. Occupational titles were coded according to the ISCO 2010 classification (29) into: professionals (ISCO 1-2) and associate professionals and service workers (ISCO 3-5). Self-rated health was assessed by a 5-point scale: good, rather good, average, rather poor, poor. Body mass index (BMI) was calculated from self-reported body weight and height as kg/m^2^ and classified according to WHO into five categories: underweight, normal, overweight, moderate obese, severe obese (30).

### Statistical analysis

The characteristics of the participants are presented as mean values and SD for the continuous variables and as frequencies and percentages for the categorical variables. Mean levels of SED, LPA, MVPA, and steps for office and remote workdays were first calculated by averaging all the available workdays per person across the workday types. The within-individual comparison in mean levels of SED, LPA, MVPA, and steps between office and remote workdays, worktime, and non-worktime was examined using linear mixed models, adjusted for age, sex, BMI, and corresponding sensor wear time during waking hours (daily, worktime or workday’s non-worktime sensor wear time according to the comparison). Workday type was included as a fixed effect. The within-subject dependency was accounted for by specifying workday as a repeated factor at the participant level. The results are given as mean estimates and mean difference estimates with their 95% confidence intervals (CI). The SAS Software 9.4 was used for the statistical analyses (SAS Institute Inc., Cary, NC).

## Results

The mean age of the study participants was 41.6 (SD 10.5, range 25–65) years. The mean number of reported weekly working hours was 37.8 (SD 3.6, range 29–50). Overall, 84% were women, 85% had a professional occupation, and 55% had a normal BMI. Of the participants, 63% had good self-reported health, 25% were living alone, and 60% did not have children <18 years of age (table 1). Measurement data included mean of 2.4 (SD 0.8) valid office workdays and 2.6 (SD 0.8) valid remote workdays. The majority (89%) of the remote workdays took place at home. The individual observations of the levels of SED, LPA, and MVPA for office and remote workdays are illustrated as boxplots in the supplementary material (www.sjweh.fi/article/4335), figure S1.

**Table 1:** Characteristics of the analytical sample of hybrid workers (N=97). [SD=standard deviation; BMI=body mass index]

	N (%)	Mean (SD)
Age, years		41.6 (10.5)
Weekly working hours ^a^		37.8 (3.6)
Women	81 (84)	
Occupation		
Professionals	82 (85)	
Associate professionals and service workers	15 (15)	
BMI classes		
Underweight	0 (0)	
Normal	53 (55)	
Overweight	31 (32)	
Moderate obese	10 (10)	
Severe obese	3 (3)	
Self-reported health ^a^		
Good	60 (63)	
Rather good	27 (28)	
Average	9 (9)	
Rather poor	0 (0)	
Poor	0 (0)	
Living alone	24 (25)	
No children <18 years old	58 (60)	

^a^ Missing N=1

Table 2 presents comparisons for daily total, worktime, and non-worktime wear time, SED, PA, and steps between office and remote workdays. On average, participants accumulated 09:58 hours of SED on office workdays and 10:42 hours of SED on remote workdays, mean difference being 44 minutes (95% CI 25–64). In addition, remote workdays included less LPA (mean difference 29 minutes, 95% CI 11–48), less MVPA (mean difference 15 minutes, 95% CI 10–20), and fewer daily steps (mean difference 1040, 95% CI 228–1852) compared to office workdays. Figure 1 illustrates the daily patterns of total PA (LPA+MVPA) during office and remote workdays. Although these two patterns were rather similar in shape, more PA was accumulated during office workdays, especially during morning hours.

**Table 2:** Within-individual comparisons for daily total, worktime, and non-worktime behaviors between office and remote workdays. Models were adjusted for age, sex, body mass index, and, daily, worktime, or non-worktime sensor wear time according to the comparison. [CI=confidence interval]

	Office workdays (minutes)		Remote workdays (minutes)		Difference between office and remote workdays (minutes)
	Mean (95% CI)		Mean (95% CI)		Mean (95% CI)
Wear time					
Daily total	972 (963–982)		961 (951–970)		-12 (-23– -0.6) ^a^
Worktime	458 (447–470)		459 (447–470)		0.4 (-15–15)
Non-worktime	495 (478–512)		493 (476–510)		-2 (-25–21)
Sedentary behavior					
Daily total	598 (567–628)		642 (612–672)		44 (25–64) ^a^
Worktime	302 (281–322)		334 (313–354)		32 (16–48) ^a^
Non-worktime	288 (272–304)		302 (272–304)		14 (3–25) ^a^
Light physical activity					
Daily total	321 (291–350)		292 (262–321)		-29 (-48– -11) ^a^
Worktime	145 (129–161)		120 (104–136)		-25 (-41– -9) ^a^
Non-worktime	172 (157–187)		166 (151–180)		-6 (-16–3)
Moderate-to-vigorous physical activity					
Daily total	49 (43–54)		33 (28–39)		-15 (-20– -10) ^a^
Worktime	13 (11–15)		6 (4–8)		-7 (-9– -5) ^a^
Non-worktime	35 (29–40)		27 (22–33)		-7 (-11– -3) ^a^
Number of steps					
Daily total	14 620 (13513–15727)		13 580 (12477–14683)		-1040 (-1852– -228) ^a^
Worktime	4715 (4316–5113)		3973 (3576–4369)		-742 (-1199– -286) ^a^
Non-worktime	9644 (8776–10513)		9296 (8427–10164)		-349 (-1033–335)

^a^ Significant difference between office and remote workdays.

When focusing on the differences within the working hours, the office worktime included 05:02 hours of SED in comparison to 05:34 hours during remote worktime (mean difference 32 minutes, 95% CI 16–48) (Table 2). Remote worktime also included less LPA (mean difference 25 minutes, 95% CI 9–41), less MVPA (mean difference 7 minutes, 95% CI 5–9), and fewer steps (mean difference 742, 95% CI 286–1199) compared to the office worktime. No differences in worktime lengths between office and remote workdays were observed (Table 2). During non-working hours, remote workdays had 14 minutes (95% CI 3–25) higher SED and 7 minutes lower MVPA (95% CI 3–11) compared to non-working hours within office workdays, whereas the amount of non-worktime LPA and steps did not differ between the workday types (table 2).

**Figure 1:**
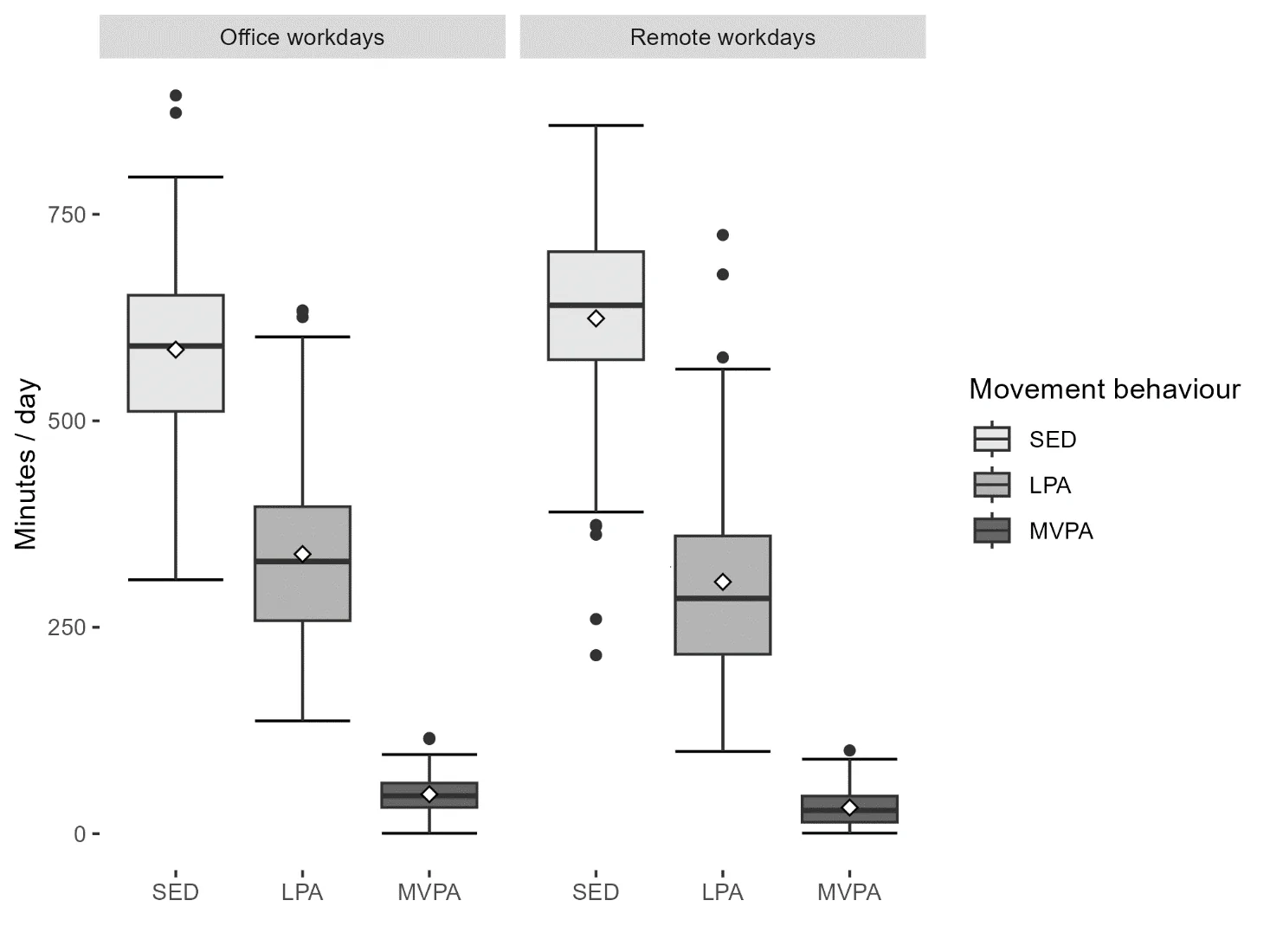
Boxplots of the observed daily minutes of sedentary time (SED), light physical activity (LPA), and moderate to vigorous physical activity (MVPA) for office and remote workdays among the hybrid workers.

**Figure 2:**
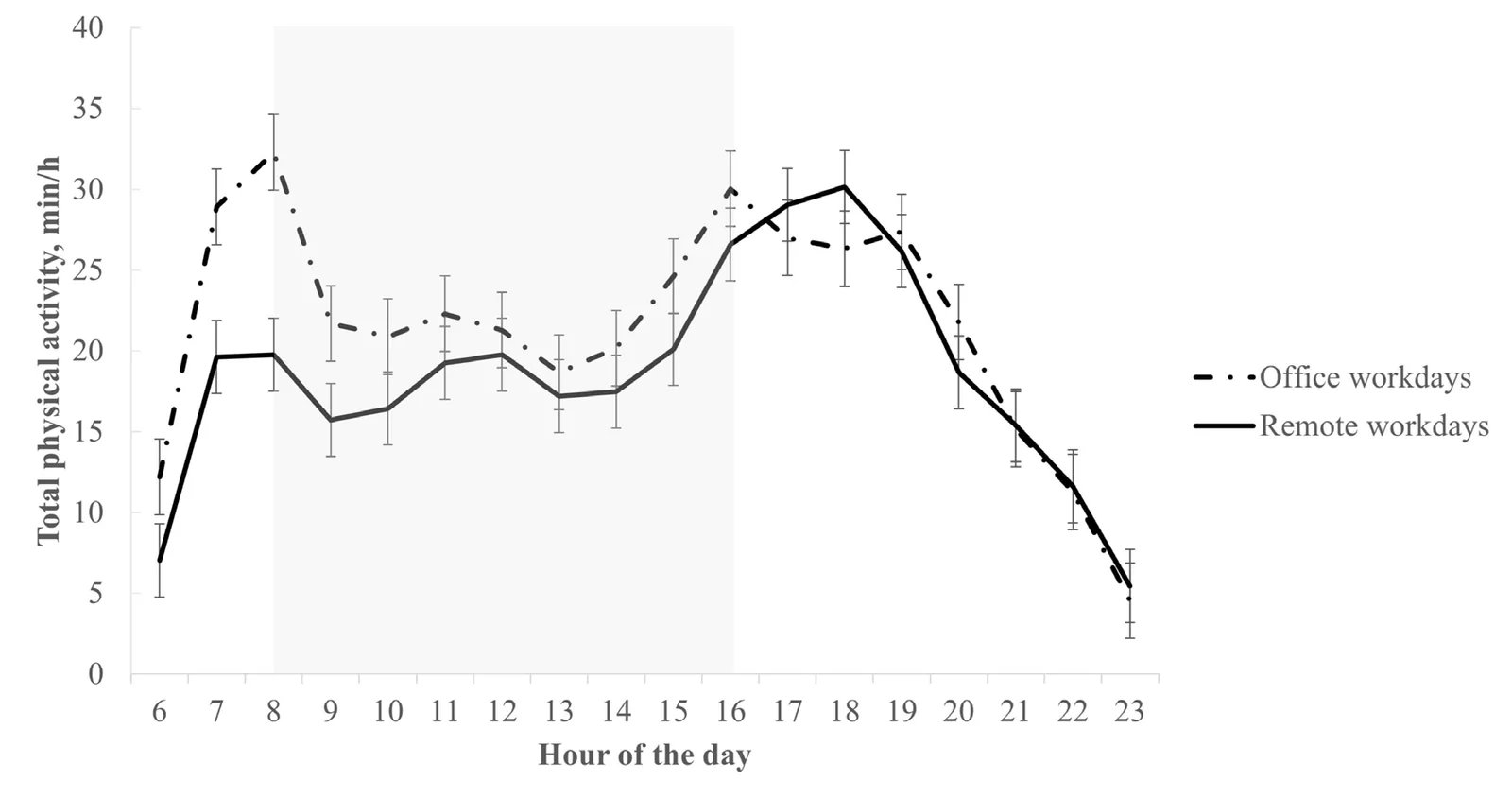
Daily patterns of total physical activity (minutes) during office and remote workdays among hybrid workers. The grey shaded area indicates typical office working hours in Finland.

## Discussion

This study aimed to compare daily total, worktime, and non-worktime PA and SED between office and remote workdays among Finnish hybrid workers during post-pandemic years. Our findings showed that participants accumulated 44 minutes more SED during remote compared to office workdays. The difference was especially seen during working hours, as remote worktime included 32 minutes more SED. Correspondingly, remote workdays comprised 29 minutes less LPA and 15 minutes less MVPA compared to office workdays. The difference in total PA was seen especially in the morning but also within the reported working hours.

Our finding of higher SED during remote versus office workdays is in line with previous accelerometer-based studies, which were conducted during the years of possible pandemic restrictions (22, 23) and during the post-pandemic period (16–18), but contradicts with pandemic-time findings from Sweden (20, 21). The 32-minute difference in worktime SED is similar to those reported from wrist- (17, 22) and thigh-worn (16) accelerometer studies among office workers. The observed 30-minute difference is also comparable to the effects reported for office-based interventions aimed at reducing worktime sitting (31), suggesting a potentially meaningful target for behavioral change. Although clinical significance cannot be determined from our study, this magnitude of difference is similar to a change considered clinically relevant in intervention studies (32).

Similarly, our findings complement the previous accelerometer-based findings among office workers of the lower LPA and MVPA levels (18, 22, 23) and lower number of steps (16, 18) when working from home. The added contribution of the current study was that we were able to examine working and non-working hours separately and observed that the difference in LPA and steps accumulated during worktime, whereas the MVPA difference was seen during both work and non-worktime. The lower non-worktime MVPA during remote versus office workdays may refer to the lack of PA related to commuting. Unfortunately, we were not able to differentiate the timing of commuting activity in our study. However, the hourly patterns showed that the difference in total PA between office and remote workdays was especially seen during the morning hours when commuting to work usually occurs. Lack of especially active commuting, which is an important way to accumulate health-enhancing PA among office workers, may therefore be the main reason for lower MVPA during remote workday’s non-worktime. Our previous findings from the WORKDAY study support this interpretation, as greater SED and lower PA during non-worktime on remote workdays, compared to office workdays, were more pronounced among active than passive commuters (33). Our current observations thus suggest that the PA that is lost due to lack of commuting is not likely to be replaced during the day when working remotely.

There are several possible explanations for higher SED and lower PA during remote working hours. The home environment, in which most of the remote work is done, may demand shorter transitions to, eg, lunch, coffee, or printer compared to office environments (13), in which these transitions usually accumulate more PA (34). The absence of sit–stand desks at home may, in turn, make it more challenging to work while standing, and limit opportunities for taking breaks from sitting, particularly during tasks requiring on-camera attendance, such as online meetings (19). Moreover, in desk-based jobs, sitting is likely to occur as an automatic by-product of carrying out work tasks (35). As the working hours at home are likely to be less structured and include fewer external interruptions, the sitting automaticity may be reinforced through increased immersion in work tasks (19, 36). Thus, prolonged sitting bouts might become longer and more frequent while working from home, particularly during concentrated work.

Previous interventions to break up worktime sitting with eg, sit-stand desks or digital prompts, have been shown to be effective in reducing SED in office environments (7, 37). Breaking up prolonged sitting has been shown to offer health benefits to eg, postprandial glucose (38, 39) and mental wellbeing, body composition, and blood pressure (4), particularly when breaks from sitting are spent being physically active (38). Decreasing worktime SED may also improve work performance, mood, and work satisfaction, and reduce musculoskeletal pain (40). However, there is still a lack of evidence on whether the office-based tools are applicable, feasible, or effective in reducing sitting and/or increasing PA at home or other remote work environments (14, 19, 37). In addition, education, social support, and modeling, eg, breaking up sitting during meetings by the managers, are suggested to play an important role in reducing SED in office settings (7) but also when working from home (19). In fact, support from co-workers has been shown to lead successful interventions in office-based studies (8), whereas support from employers or managers may be more crucial when working remotely (41).

Taken together, there are multiple aspects that need to be taken into consideration in future interventions and strategies aiming to increase PA and decrease SED among remote workers. Successfully reducing worktime sitting is likely to require multi-component interventions, with focus on behavioral self-regulation, environmental opportunities, and social norms and practices (36, 41). Furthermore, rather than focusing solely on reducing SED (with standing), future interventions should aim to increase movement, eg, with active breaks (39) to increase light PA (42) or MVPA (43) for health benefits. It is noteworthy that promoting MVPA has proven to be difficult in office-based interventions (6), suggesting that, overall, new strategies to increase MVPA among office employees are needed. Working from home might hold potential for promoting MVPA, given that common barriers to increase MVPA at the office, such as workplace culture and lack of flexibility (44), may not be as relevant at home. At best, more flexible working hours, absence of social barriers, and eg, easier access to outdoors when working from home could provide opportunities for longer and higher-intensity active breaks or to increase time in recreational or household activities (45). This could further serve to replace the PA that appears to be lost due to lack of (active) commuting.

A major strength of our study is that it was conducted among post-pandemic hybrid workers, enabling within-individual comparison of PA and SED between office and remote workdays after pandemic restrictions. With daily logs, we were able to identify daily working hours to study worktime PA and SED apart from the non-working hours. As a methodological strength, we used 24/7 thigh-worn sensors to sensitively capture postural changes required for the separation of different activities (25).

The generalizability of our results may be limited because the participants were recruited primarily from a university setting, where the level of education and work-related autonomy may be relatively high compared to other sectors and occupations (46). Consequently, our sample was rather homogenous and consisted predominantly professional or associate professionals. However, role-specific differences also exist within the academic environment. For example, researchers, teachers, and administrative personnel may differ in the nature of their work tasks and the level of workday autonomy, with implications for sitting time and possibilities to break up sitting (47). Nevertheless, detailed data on the nature of work tasks were not available, precluding further examinations of these differences.

Women were over-represented in our study sample compared with the overall university workforce (84% versus 58%). Also, the average number of steps per day measured in our study was almost double the national average (48). Together, these characteristics suggest potential selection bias, which may limit the transferability of the findings to other occupational sectors, as well as to non-Nordic countries and cultural contexts. However, given that the current sample was highly educated, predominantly female, and highly physically active, it is plausible that the differences between office and remote workdays may be even more pronounced in less active populations that are more representative of the workforce at large.

### Concluding remarks

Remote workdays included lower PA and higher SED than office workdays among post-pandemic hybrid workers. The differences were mostly accrued in the morning and during working hours. However, remote work with more flexibility may provide opportunities for increasing daily PA. Therefore, these findings call for actions to increase PA during remote workdays in addition to the commonly recommended strategies to reduce worktime SED.
